# Peto’s paradox revisited (revisited, revisited, revisited, and revisited yet again)

**DOI:** 10.1073/pnas.2502696122

**Published:** 2025-03-31

**Authors:** Vincent J. Lynch

**Affiliations:** ^a^Department of Biological Sciences, University at Buffalo, State University at New York, Buffalo, NY 14260

**Fig. 1.**
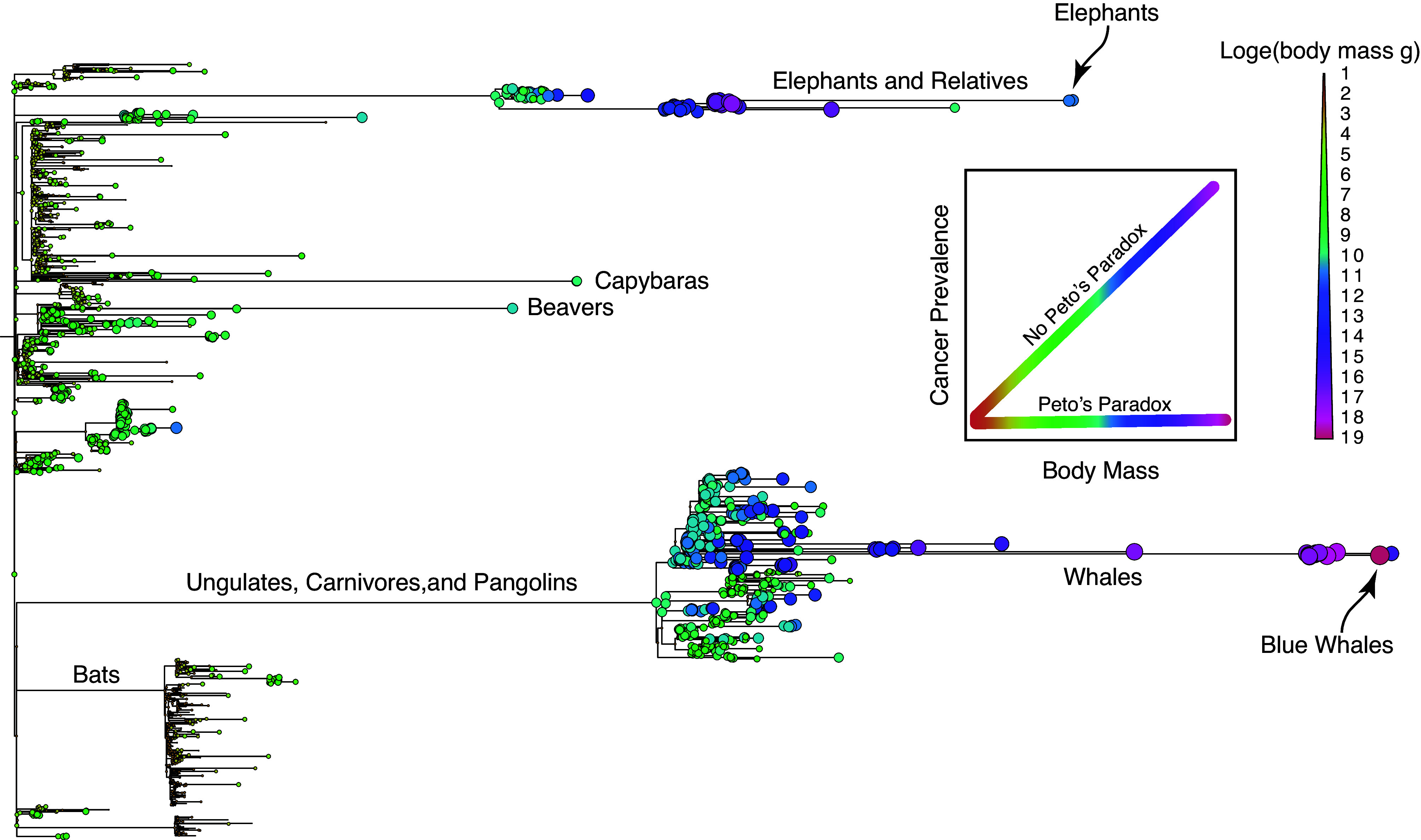
Peto’s paradox and body size evolution in mammals. Phylogeny of Eutherian mammals with branch lengths scaled by log_2_ change in body size. Circles at tip branches represent the log_2_ body mass of each species, while circles at internal nodes illustrate the log_2_ ancestral state reconstruction of body mass inferred from the StableTraits model (13). Lineages with long branches had rapid body mass evolution and may have lower cancer prevalence than expected based on their body size.

Cancer, a scourge of multicellular animals, arises when a single cell acquires the right combination of mutations to go rogue. These cancerous cells bypass the body’s safeguards, manipulating the communal structure of cells, tissues, and organ systems to extract resources that enable their unchecked proliferation. Over time, this exploitation weakens the system, ultimately threatening the survival of the whole organism. This raises some intriguing questions: How can extremely large animals, which contain an enormous number of cells, survive when it only takes one rogue cell to become cancerous? Similarly, if it is merely a matter of time before a cell hits upon the right combination of mutations to turn cancerous, how do animals with exceptionally long lifespans and thus a long time to acquire cancer-causing mutations exist? Within species, these expectations hold—taller humans have a higher prevalence of cancer than shorter ones (1), and the prevalence of cancer increases as we age (2). However, there is (apparently) no correlation between cancer prevalence and either lifespan or body size across species. This apparent lack of an effect of body mass and lifespan on cancer prevalence became known as Peto’s paradox (3–5), but as Peto noted, “it could more appropriately have become known as Doll’s dilemma (6) or Cairns’ conundrum (7), as both of them had emphasized how important it was to understand this properly” (5). It is particularly unexpected because the relationship between age and cancer is foundational for the multistage model of carcinogenesis (2, 8–11). In this issue of PNAS, Butler et al. reexamine Peto’s paradox and find no paradox at all; larger species have a higher cancer prevalence than smaller ones (12) (Fig. 1).

Peto helpfully illustrates the paradox (5): the prevalence of many cancers increases proportionally to the sixth power of age (i.e., for most nonpediatric cancers, five or six mutations are necessary for transformation). Thus, a simplified multistage cancer model predicts that a species’ intrinsic cancer risk (*K*) can be approximated with the formula *K* ≈ *Dt*^6^, where *D* is the maximum body size and *t* is the maximum lifespan (5); this sixth-power formula relating intrinsic cancer risk to lifespan means that doubling maximum lifespan does not double intrinsic cancer risk. Instead, it increases risk by six twofold differences (2^6^) or a 64-fold increase in risk, whereas doubling maximum body mass doubles cancer risk (5). For example, the prevalence of cancer in elephants, the largest living land mammal, and rock hyraxes, the closest living relative of elephants, is ~4.5% (14, 15). Therefore, for hyraxes and elephants to have the same cancer prevalence, the intrinsic cancer risk associated with lifespan difference must be 13,333-fold (1.66 × 6 ≈ 10 powers of 2) smaller in elephants, and the intrinsic cancer risk associated with body size differences must be ~1,024-fold (4,800 kg/3.6 kg) smaller in elephants; combining these 13,333-fold and 1,024-fold reductions intrinsic cancer risk means that elephants are ~13.37 million times less susceptible to cancer than hyraxes. These data are consistent with Peto’s paradox, but is there a paradox at higher taxonomic levels and across the classes of tetrapods?

At least four prior studies have systematically investigated the relationship between cancer, body size, and lifespan: 1) Abegglen et al. (14) surveyed 832 necropsy reports from 36 mammalian species and found no correlation between cancer mortality and either body size or lifespan; 2) Vincze et al. (16) surveyed 110,148 individuals, including 11,148 necropsy reports, from 191 mammalian species and found no correlation between cancer mortality risk or cumulative incidence of cancer mortality and body size or lifespan; 3) Bulls et al. (15) surveyed 9,631 necropsy reports from 1,060 tetrapod species and found that body mass and lifespan were positively correlated with cancer in amphibians but not in other tetrapods; 4) Compton et al. (17) surveyed 16,049 necropsy reports from 292 tetrapod species and found no correlation between body mass and lifespan cancer prevalence. In contrast, they identified a positive correlation between body mass and cancer prevalence in mammals when they adopted the analytical framework proposed by Bulls et al. (15).

One of the challenges in rigorously testing Peto’s paradox is determining the appropriate method to use. Many epidemiological studies utilize logistic regression to predict the risk of developing specific diseases, such as cancer, based on observed patient characteristics, including age, sex, and body mass, among others. However, logistic regression, which was used by Abegglen et al., fails to account for the nonindependence of observations in phylogenetic data. For instance, the observation that four species share a particularly low prevalence of cancer does not represent a dataset of four; these species share a common ancestor. If that ancestor evolved reduced cancer prevalence, then the number of observations is one rather than four. Therefore, logistic regression is not suitable for comparisons between species.

The method most commonly used to test for correlations across species while accounting for the nonindependence of data due to common ancestry is phylogenetic generalized least squares (PGLS) regression (18). However, PGLS regression, which was used by Vincze et al., and a modified version of PGLS used by Compton et al., is unsuitable for testing Peto’s paradox because cancer prevalence data are proportional, i.e., bounded between zero and one, and is not a normally distributed continuous variable. Cancer prevalence follows a binomial distribution and is heavily skewed toward zero, as observing a necropsy with cancer is rare in small sample sizes, such as those for zoo species. Therefore, the normality assumption of PGLS regression is violated. To test Peto’s paradox, Bulls et al. introduce a different method, generalized estimating equations (GEE) (19), which directly uses individual counts for each species, accommodates binomial distributions, and skewness toward zero.

Butler et al. present an alternative method to test Peto’s paradox: a multivariate phylogenetic generalized linear mixed model (MPGLMM) (18). Like GEE, MPGLMM models each species’ observed number of malignancies rather than the species-specific proportions. Unlike GEE, which uses a binomial distribution, it uses Poisson regression. To test Peto’s paradox, they fit an MPGLMM in which cancer depended on the number of necropsies and body size in a single model. Like Bulls et al., they found that body mass was positively correlated with cancer in amphibians. Unlike all previous studies, they found a positive correlation between body mass and cancer prevalence in the other tetrapod classes—larger species had a higher cancer prevalence than smaller species. Remarkably, birds and mammals that evolved large body sizes rapidly have lower rates of cancer prevalence relative to their body mass than expected, although they still demonstrate a positive correlation within those groups. Therefore, in a strict sense, these data indicate that Peto’s paradox is false. Nevertheless, a more relaxed interpretation—that cancer is less prevalent in large-bodied species than one would expect based on their body size—still holds. Thus, at least for now, comparative oncologists can have their cake and eat it, too.

These contrasting results have significant implications for comparative oncology studies across species, including understanding the origins, treatment, and prevention of human cancers. Previous studies, including our own, selected large species, such as elephants (20–22) and whales (23, 24), as well as small species with surprisingly long lifespans, like the famously cancer-resistant naked mole rats (25) and bats (26, 27), a priori because of their size and longevity, suggesting that they must have evolved ways to skirt the paradox. While exact numbers are challenging to estimate, Wikipedia suggests, without referencing supporting evidence, that there are over 65,000 vertebrate species. Which of these have solved the paradox or have found ways around it? Are there species that have evolved mechanisms to evade cancer or those that always succumb to it? How can we identify them, and what can we learn from them that might benefit human health? Identifying these species requires collecting data on cancer prevalence in very many species. So far, we have surveyed this data from only about 1.3% of them, primarily those living in zoos. It is essential to have this data, but how do we analyze and interpret it? Methods matter.

In PNAS, Butler et al. reexamine Peto’s paradox and find no paradox at all; larger species have a higher cancer prevalence than smaller ones (12).

So far we have five studies (12, 14–17), four methods, three types of data, two outcomes (support and contradiction), and one paradox that is either true or false. What is to be done? Argue. Argumentation is essential for scientific progress, as it forces us to concentrate on the critical aspects of phenomena to be studied and define the methods and the types of data to do so. Scientific advancement is occurs through debates over studies with contradictory results. It is a good time to argue over a paradox!
